# Efficacy and safety of telitacicept in systemic lupus erythematosus: a single-center, retrospective, real-world study

**DOI:** 10.3389/fimmu.2026.1890148

**Published:** 2026-07-28

**Authors:** Li Yang, Jiayu Liu, Jinmei Zou, Jing Yang

**Affiliations:** 1 Department of Rheumatology, Mian Yang Central Hospital, School of Medicine, University of Electronic Science and Technology of China, Mianyang, China; 2 Department of Emergency Medicine, Mian Yang Central Hospital, School of Medicine, University of Electronic Science and Technology of China, Mianyang, China

**Keywords:** efficacy, real-world, safety, systemic lupus erythematosus, telitacicept

## Abstract

**Objective:**

To explore the real-world efficacy and safety of telitacicept in patients with systemic lupus erythematosus (SLE).

**Methods:**

This retrospective study included SLE patients treated with telitacicept for at least 3 months at Mianyang Central Hospital between January 2022 and December 2025. Baseline characteristics and clinical outcomes at 12, 24, and 52 weeks were assessed.

**Results:**

A total of 139 patients were enrolled in this study. The SLE Disease Activity Index 2000 (SLEDAI-2K) score significantly decreased from a baseline of 11.42 ± 4.68 to 4.623 ± 3.65, 2.42 ± 2.98, and 1.48 ± 2.58 at 12, 24, and 52 weeks, respectively (*P* < 0.0001). The anti-dsDNA positivity rate declined from 35.25% at baseline to 7.94% by week 52, accompanied by an upward trend in complement 3 (C3) and complement 4 (C4) levels. Among the 74 patients with lupus nephritis, the baseline 24-hour urine protein was 1.59 ± 1.68 g/24h, which significantly decreased to 1.12 ± 1.41 g/24h, 0.42 ± 0.46 g/24h, and 0.39 ± 0.64 g/24h at 12 weeks, 24 weeks, and 52 weeks, respectively (*P* < 0.001). The serum albumin level significantly increased (*P* < 0.0001), while the serum estimated glomerular filtration rate (eGFR) showed significant reductions (*P* < 0.05). Furthermore, white blood cell and platelet count also improved significantly in patients with leukopenia and thrombocytopenia (P < 0.0001). The mean daily glucocorticoids dose (equivalent to prednisone) was successfully tapered from 39.98 ± 10.09 mg/d at baseline to 18.35 ± 7.87 mg/d at 12 weeks, 9.752 ± 4.72 mg/d at 24 weeks, and 5.69 ± 2.95 mg/d at 52 weeks (*P* < 0.0001). Adverse events occurred in 28 (20.14%) patients, with upper respiratory tract infections being the most common (53%), and no serious adverse events occurred.

**Conclusion:**

In this real-world study, telitacicept combined with conventional therapy was associated with improvements in disease activity and organ functions in SLE with favorable safety.

## Introduction

Systemic lupus erythematosus (SLE) is a chronic, highly heterogeneous autoimmune disease that predominantly affects women of childbearing age, with a reported male-to-female ratio of 1:9 (1). The current incidence of SLE is 134 per 100,000 among Caucasians and Europeans (2), while in China, it ranges from 30 to 70 per 100,000 (3). Although the exact pathogenesis of SLE remains unclear, genetic susceptibility, environmental triggers, hormonal influences, and immune dysregulation are widely recognized as contributing factors (4, 5). Studies indicate that B cells participate in the pathogenesis of SLE through antigen presentation and production of autoantibodies and cytokines. Key pathways involved in aberrant B cell activation include the toll-like receptor (TLR) pathway, B cell activating factor (BAFF) stimulation, and B cell receptor (BCR) mediated activation (6). Furthermore, imbalance in B cell subsets are closely related to disease activity and organ damage in SLE (7, 8). Clinical manifestations of SLE are complex and multi-systemic, potentially involving the skin, mucosa, joints, muscles, kidneys, hematologic system, nervous system, lungs, and gastrointestinal tract. Without timely intervention, the disease can cause irreversible tissue and organ damage, and even lead to death. Currently, no clinical cure exists for SLE. The primary therapeutic goals are to minimize organ damage, delay disease progression, improve patient outcomes, and prevent relapse (9). Conventional management relies on glucocorticoids, antimalarial drugs, and immunosuppressants. Although these therapies can reduce disease severity, their efficacy remains limited by low response rates, high recurrence rates, and notably side effects. Consequently, current guidelines recommend biological agents for patients with refractory or relapsed SLE. These targeted therapies have been shown to significantly improve complete and partial response rates, reduce disease activity and recurrence, and facilitate glucocorticoid tapering (10).

In recent years, several targeted drugs focusing on SLE pathogenesis have been developed, including anti-CD20 monoclonal antibodies (rituximab, ocrelizumab), anti-CD22 monoclonal antibodies (epalizumab), anti-B cell stimulating factors (belimumab, etanercept). Telitacicept is a novel fully human TACI-Fc fusion protein constructed by linking the extracellular domain of TACI to the Fc fragment of IgG1 antibody. Distinct from single-target agents, telitacicept simultaneously targets both B lymphocyte stimulator (BlyS) and a proliferation-inducing ligand (APRIL). By blocking BlyS, it inhibits B cell development and maturation, thereby slowing long-term disease progression. Meanwhile, blocking APRIL prevents mature B cells differentiating into plasma cells and reduces the production of pathogenic autoantibodies, effectively controlling disease activity (11, 12). Although telitacicept was approved for SLE treatment in China in 2021 following robust Phase III clinical trial results (13), its clinical performance and safety remain to be characterized. Therefore, this study aimed to explore the efficacy and safety of telitacicept in real-world.

## Patients and methods

### Patients

This study retrospectively screened patients with SLE admitted to Mianyang Central Hospital between January 2022 and December 2025. Eligible patients should meet the 1997 American College of Rheumatology classification criteria or the 2019 European Alliance of Associations for Rheumatology/American College of Rheumatology classification criteria and had received telitacicept for at least three months. Patients who had used other B cell targeted biological within 3 months before enrollment were excluded. In total, 139 SLE patients were enrolled in this study.

### Study design

This study was a single-center, retrospective, uncontrolled observational study. The primary endpoint was the change in SLE Disease Activity Index 2000 (SLEDAI-2K) score at 24 weeks. Secondary endpoints included changes in SLEDAI-2K at 12 weeks and 52weeks, along with changes in anti-double-stranded DNA (anti-dsDNA), complements, immunoglobulin, 24-hour urine protein, white blood cells (WBCs), platelets (PLT) at 12 weeks, 24 weeks and 52 weeks. The reduction in daily glucocorticoid dosage over the follow-up period was evaluated as an indicator of therapeutic efficacy. Additionally, safety was evaluated by monitoring and recording the occurrence of adverse events during the study.

### Data collection

Comprehensive demographic, clinical, and laboratory data were collected for all patients at baseline and throughout follow-up. Baseline characteristics included sex, age, and disease duration. Clinical assessments consisted of the SLEDAI-2K score and identification of the primarily affected organs. Laboratory indicators include antinuclear antibody (ANA), anti-dsDNA antibody, complement 3(C3) and complement 4 (C4), and immunoglobulins (IgG, IgM, and IgA). For patients with renal involvement, 24-hour urine protein quantification, urine albumin/creatinine ratio (UACR), serum albumin, serum creatinine, and estimated glomerular filtration rate (eGFR) were recorded. For those with hematological system involvement, white blood cell and platelet counts were recorded. Additionally, detailed treatment profiles, including dosages and durations of glucocorticoids, immunosuppressants, hydroxychloroquine, and biologic agents were also documented.

### Statistical analysis

Statistical analyses were performed using SPSS 22.0 and GraphPad Prism 10. Continuous variables were presented as mean ± standard deviation (SD), while categorical variables were expressed as percentages. Missing data were handled using multiple imputation. Analysis of variance (ANOVA) was applied for comparisons across multiple time points. Paired t-tests were used for pairwise comparisons between baseline and 12, 24, and 52 weeks. A two-sided *P* < 0.05 was considered statistically significant.

This study was approved by the Ethics Committee of Mianyang Central Hospital, School of Medicine, University of Electronic Science and Technology of China (S20260398-01). Written informed consent before telitacicept treatment was obtained from all patients. All patient identifiers were removed before analysis.

## Results

### Baseline characteristics of included patients

A total of 139 patients were enrolled, 94.96% of whom were female. The mean age was 41.46 years, with 136 adults and three patients under 18 years. Mean disease duration was 5.935 ± 5.512 years. Of these, 38 patients had a disease duration of less than 1 year (including 16 newly diagnosed cases), and 39 patients had a disease history exceeding 10 years. The mean SLEDAI-2K score was 11.42 (range: 4-26). There were 74 patients (53.24%) with nephritis, 67 patients (48.20%) with hematological involvement, and 48 patients (34.53%) with joint and muscle damage. Low complement levels were observed in 112 patients (80.58%), and 49 patients (35.25%) tested positive for anti-dsDNA antibodies. All patients initially received glucocorticoid therapy, and 137 patients (98.56%) taking a daily dose exceeding 7.5 mg. Hydroxychloroquine was given to 132 patients (94.96%). Cyclosporine was the most commonly used immunosuppressant (45, 32.37%), followed by mycophenolate mofetil (40, 28.78%) and tacrolimus (37, 26.62%). For telitacicept, 53% of patients received 80 mg once weekly (qw), and 47% received 160 mg qw. There was no statistically difference in the SLEDAI-2K scores at baseline between the two groups. All 139 patients completed the 12-week follow-up, 101 continued treatment for six months and completed the 24-week follow-up, and 63 completed the 52-week follow-up (**Table 1**). Reasons for discontinuation telitacicept and loss to follow-up included economic constraints and difficulties with drug storage and administration. After six months, some patients stopped telitacicept because their condition had stabilized.

**Table 1:** The baseline main characteristics of the included patients.

Demographic/characteristic	All, n=139
Age	
Mean (year)	41.46
<18 years old, n (%)	3 (2.16)
≥18 years old, n (%)	136 (97.84)
Female, n (%)	132 (94.96)
Disease duration	
All, mean ± SD (year)	5.935 ± 5.512
<1 year, n (%)	38 (27.34)
1 to 10 years, n (%)	62 (44.60)
>10 years, n (%)	39 (28.06)
SLEDAI-2K scores, mean ± SD	11.42 ± 4.697
SLEDAI-2K scores, n (%)	
Mild (0–6)	17 (12.23)
Moderate (7–12)	75 (53.96)
Severe (>12)	47 (33.81)
Clinical manifestations by organ system involvement, n (%)	
CNS	4 (2.88)
Vasculitis	28 (20.14)
Musculoskeletal	48 (34.53)
Renal	74 (53.24)
Hair loss or skin and mucosa	66 (47.48)
Serosa	15 (10.79)
Immune abnormalities	119 (85.61)
Fever	25 (17.99)
Abnormal blood system	67 (48.20)
ANA positive, n (%)	136 (97.84)
Anti-dsDNA positive, n (%)	49 (35.25)
Decreased C3, n (%)	103 (74.10)
Decreased C4, n (%)	86 (61.87)
C3, mean ± SD (g/L)	0.56 ± 0.26
C4, mean ± SD (g/L)	0.11 ± 0.07
Glucocorticoid (prednisone or methylprednisolone), n (%)	139 (100)
Glucocorticoid (prednisone or equivalent), mean ± SD (mg/day)	39.98 ± 10.09
Glucocorticoid > 7.5 mg/day, n (%)	137 (98.56)
Immunosuppressant, n (%)	
All	124 (89.21)
Ciclosporin	45 (32.37)
Methotrexate	5 (3.60)
Mycophenolate mofetil	40 (28.78)
Cyclophosphamide	5 (3.60)
Tacrolimus	37 (26.62)
Leflunomide	5 (3.60)
Azathioprine	1 (0.72)
Iguratimod	1 (0.72)
Tofacitinib	3 (2.16)
Antimalarial drug, n (%)	
Hydroxychloroquine	132 (94.96)
Telitacicept dose	
160mg, n (%)	64 (46.04)
SLEDAI-2K scores, mean ± SD	11.19 ± 4.552
80mg, n (%)	75 (53.96)
SLEDAI-2K scores, mean ± SD	11.70 ± 4.882
Patients number in follow-up	
12 weeks, n (%)	139 (100)
24 weeks, n (%)	101 (72.66)
52 weeks, n (%)	63 (45.32)

SLEDAI-2K, systemic lupus erythematosus disease activity index 2000. CNS, central nervous system. ANA, antinuclear antibody. Anti-dsDNA, anti-double-stranded DNA. C3, complement 3. C4, complement 4.

### Efficacy outcomes

**Figure 1:**
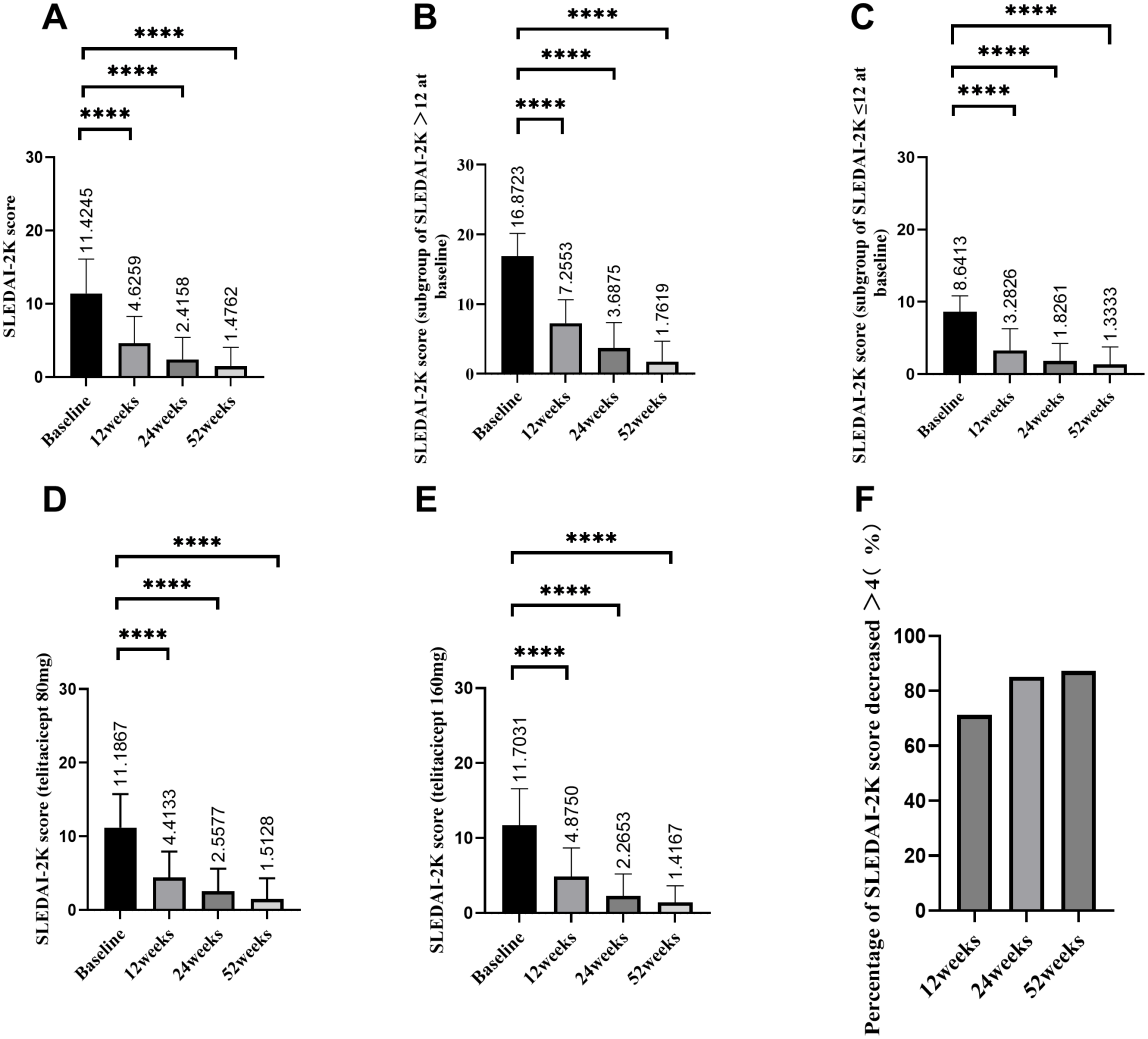
SLEDAI-2K (systemic lupus erythematosus disease activity index 2000) score shown as mean values. **(A)** SLEDAI-2K scores at baseline, 12 weeks, 24 weeks, and 52 weeks; **(B)** SLEDAI-SK scores in the subgroup of SLEDAI-2K > 12; **(C)** SLEDAI-2K scores in the subgroup of SLEDAI-2K ≤ 12; **(D)** SLEDAI-SK scores in telitacicept 80mg group; **(E)** SLEDAI-SK scores in telitacicept 160mg group; **(F)** percentage of SLEDAI-2K decreased > 4 at 12 weeks, 24 weeks, and 52 weeks. ****P <.0001 vs. baseline.

The baseline SLEDAI-2K score was 11.42 ± 4.679, which significantly decreased to 4.626 ± 3.652, 2.416 ± 2.984, and 1.476 ± 2.583 at 12, 24, and 52 weeks, respectively (*P* < 0.0001) (**Figure 1A**). Consistent improvements were observed across subgroup analyses. In the severe active group (baseline SLEDAI-2K > 12), the SLEDAI-2K score dropped significantly from 16.87 ± 3.275 at baseline to 7.255 ± 3.391, 3.688 ± 3.685, and 1.762 ± 2.914 at weeks 12, 24, and 52, respectively (*P* < 0.0001) (**Figure 1B**). In the mild to moderate active group (baseline SLEDAI-2K ≤ 12), the SLEDAI-2K significantly declined from 8.641 ± 2.207 at baseline to 3.283 ± 3.003 at 12 weeks, 1.826 ± 2.407 at 24 weeks, and 1.333 ± 2.426 at 52 weeks, respectively (*P* < 0.0001) (**Figure 1C**). We further stratified patients by telitacicept dosage (80 mg and 160 mg) and evaluated SLEDAI-2K scores at each follow-up visit. In both groups, scores were significantly lower than baseline at weeks 12, 24, and 52 (**Figures 1D, E**). Additionally, the proportion of patients achieving a ≥4 point reduction in SLEDAI-2K score reached 71.22%, 85.14%, and 87.30% at 12, 24, and 52 weeks, respectively (**Figure 1F**).

### Immune parameters

**Figure 2:**
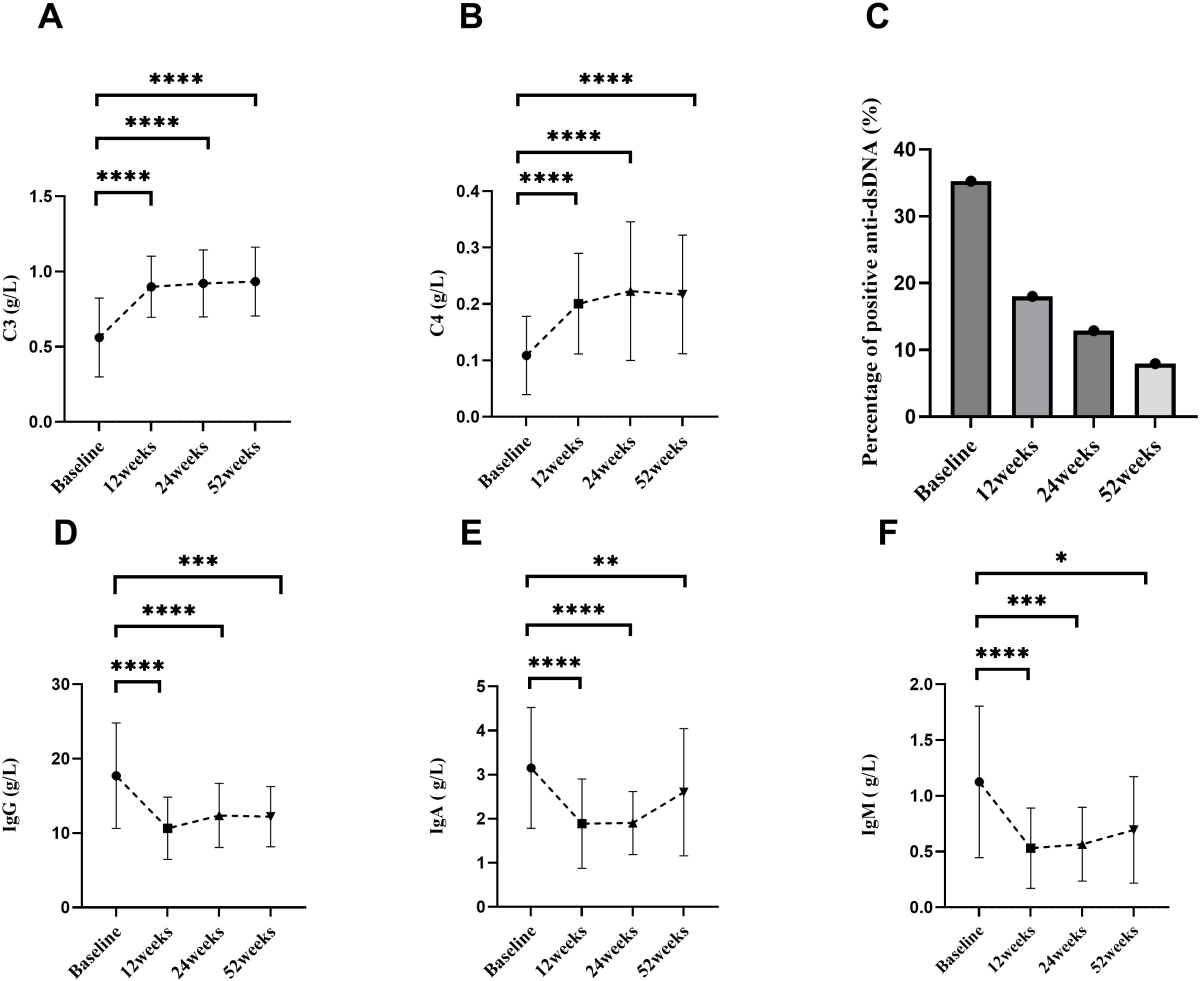
Immune parameters at baseline,12 weeks, 24 weeks, and 52 weeks. **(A)** C3 (complement 3), **(B)** C4 (complement 4), **(C)** percentage of positive anti-dsDNA, **(D)** IgG (immunoglobulin G), **(E)** IgA (immunoglobulin A), **(F)** IgM (immunoglobulin M). *P <.05 vs. baseline; **P <.01 vs. baseline; ***P <.001 vs. baseline; ****P <.0001 vs. baseline.

Significant improvements in immunological parameters were observed throughout follow-up. Baseline C3 levels were 0.56 ± 0.26 g/L, and increased significantly to 0.90 ± 0.20 g/L at 12 weeks, 0.92 ± 0.22 g/L at 24 weeks, and 0.93 ± 0.23 g/L at 52 weeks (*P* < 0.0001) (**Figure 2A**). C4 levels rose from 0.11 ± 0.07 g/L at baseline to 0.20 ± 0.09 g/L, 0.22 ± 0.12 g/L, and 0.22 ± 0.10 g/L at the corresponding time points (*P* < 0.0001) (**Figure 2B**). Concurrently, the anti-dsDNA positivity rate steadily declined, falling from 35.25% at baseline to 18.00% at 12 weeks, 12.88% at 24 weeks, and 7.94% at 52 weeks (**Figure 2C**). Additionally, serum immunoglobulin levels (IgG, IgA, IgM) gradually decreased over the 52-week period while remaining within normal ranges (**Figures 2D–F**).

### Renal outcomes

**Figure 3:**
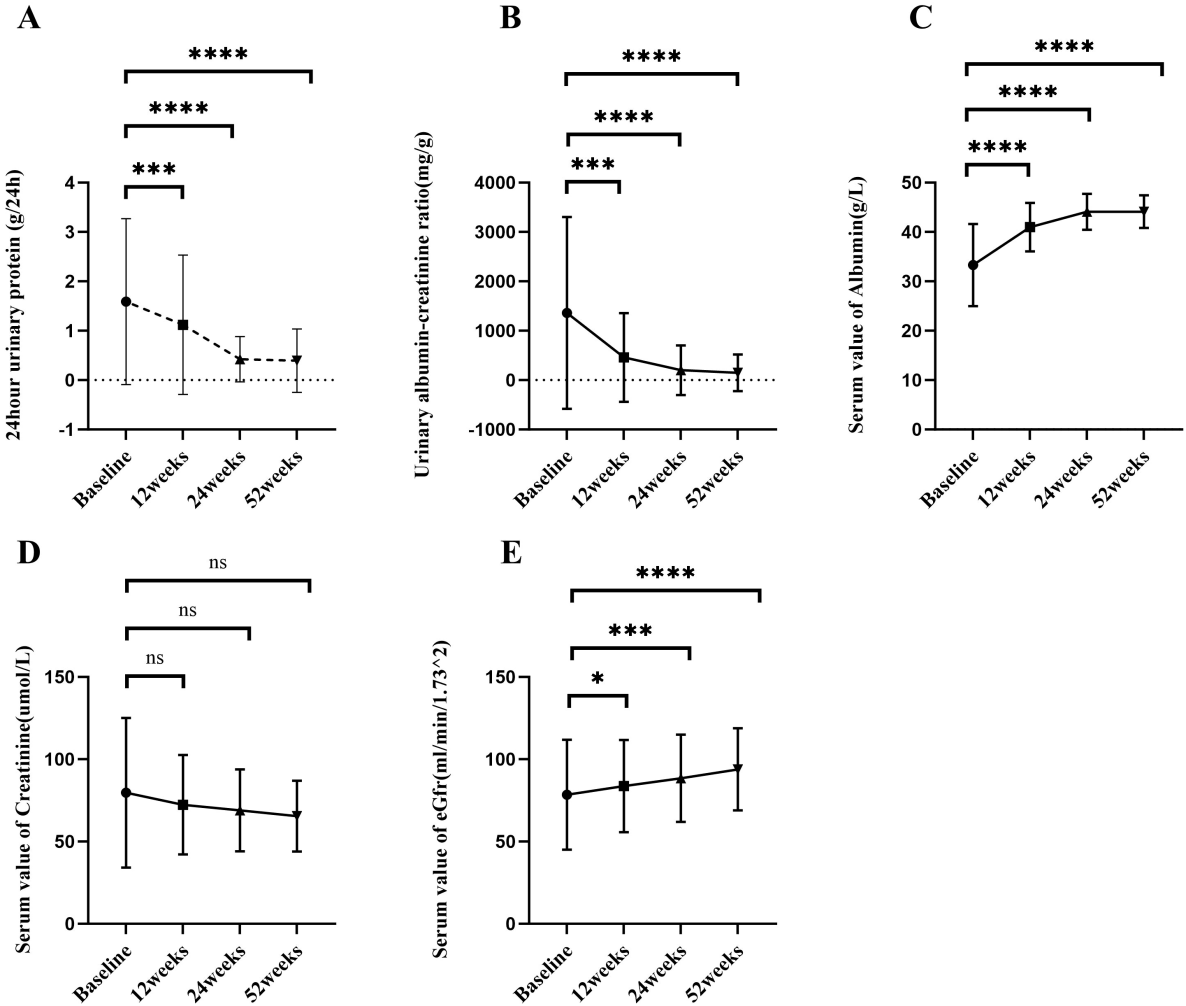
Nephritis-related parameters at baseline, 12 weeks, 24 weeks, and 52 weeks. **(A)** 24-hour urinary protein, **(B)** urine albumin/creatinine ratio (UACR), **(C)** serum value of albumin, **(D)** value of creatinine, **(E)** eGFR (estimated glomerular filtration rate). *P <.05 vs. baseline; **P <.01 vs. baseline; ***P <.001 vs. baseline; ****P <.0001 vs. baseline; NS means no significant.

There were 74 patients (53.24%) diagnosed with nephritis, 25 of them underwent renal biopsy, and most were classified as type IV+V. Renal dysfunction occurred in 38 patients, 24 acute and 14 chronic. Hydroxychloroquine was administered to 69 patients, and 68 received immunosuppressive, of which cyclosporine, tacrolimus, and mycophenolate mofetil were the most commonly used (**Table 2**). 24-hour urine protein quantification decreased significantly from baseline (1.59 ± 1.68 g/24h) to 1.12 ± 1.41 g/24h at 12 weeks (*P* = 0.0005), 0.42 ± 0.46 g/24h at 24 weeks (*P* < 0.0001), and 0.39 ± 0.64 g/24h at 52 weeks (*P* < 0.0001) (**Figure 3A**). Similarly, UACR exhibited a significant and sustained decline from baseline at all follow-up time points (*P* < 0.0001) (**Figure 3B**). Additionally, serum albumin level improved from 33.29 ± 8.30 (g/L) at baseline to 40.99 ± 4.91(g/L) at 12 weeks, 44.09 ± 3.64 (g/L) at 24 weeks, and 44.11 ± 3.32 (g/L) at 52 weeks (*P*<0.0001) (**Figure 3C**). Regarding renal function, creatinine decreased from baseline (79.68 ± 45.43μmol/L) to 72.35 ± 30.19μmol/L at 12 weeks (*P* = 0.5627), 68.95 ± 24.90μmol/L at 24 weeks (*P* = 0.3162), and 65.48 ± 21.49μmol/L at 52 weeks (*P* = 0.1374), although these differences were not statistically significant, the values trended downward overall (**Figure 3D**). Concurrently, eGFR significantly increased from a baseline of 78.40 ± 33.38 (ml/min/1.73 m²) to 83.65 ± 27.98 (ml/min/1.73 m²) (*P* = 0.0110), 88.41 ± 26.49 (ml/min/1.73 m²) (*P* = 0.0004), and 93.86 ± 24.97 (ml/min/1.73 m²) (*P* < 0.0001) at 12, 24, and 52 weeks, respectively (**Figure 3E**).

**Table 2:** The main characteristics of the patients with lupus nephritis.

Characteristics of lupus nephritis patients	All, n=74
Renal histopathological classification (ISN/RPS Class), n (%)	
All	25 (33.78)
Class III	3 (12.00)
Class IV	5 (20.00)
Class V	4 (16.00)
Class III+V	6 (24.00)
Class IV+V	7 (28.00)
Abnormal renal function, n (%)	38 (51.35)
Acute renal dysfunction	24 (63.16)
Chronic renal dysfunction	14 (36.84)
Immunosuppressant, n (%)	
All	68 (91.89)
Ciclosporin	14 (20.59)
Tacrolimus	14 (20.59)
Mycophenolate mofetil	14 (20.59)
Cyclophosphamide	5 (7.35)
Ciclosporin + Mycophenolate mofetil	8 (11.76)
Tacrolimus + Mycophenolate mofetil	8 (11.76)
Leflunomide	4 (5.88)
Methotrexate	1 (1.47)
Antimalarial drug, n (%)	
Hydroxychloroquine	69 (93.24)

ISN/RPS, international society of nephrology/renal pathology society. Class III, focal lupus nephritis. Class IV, diffuse lupus nephritis. Class V, membranous lupus nephritis.

### Hematologic outcomes

**Figure 4:**
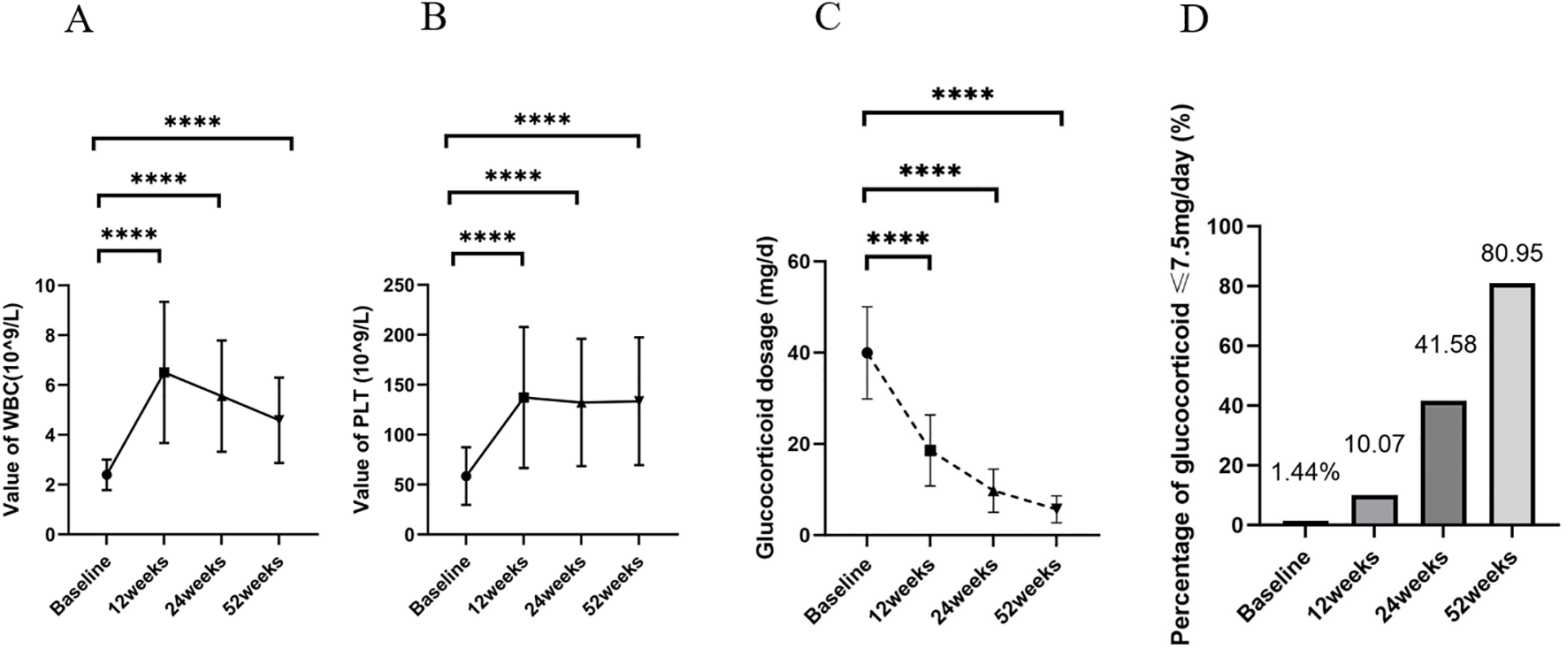
Hematological system -related parameters and glucocorticoid dose at baseline, 12 weeks, 24 weeks, and 52 weeks. **(A)** WBC (white blood cell). **(B)** PLT (platelet). **(C)** Glucocorticoid dose at baseline,12 weeks, 24 weeks, and 52 weeks. **(D)** Percentage of glucocorticoid ≤ 7.5mg/d at baseline,12 weeks, 24 weeks, and 52 weeks. ****p <.0001 vs. baseline.

A total of 67 patients (48.20%) was diagnosed with hematological system damage. At baseline, 42 patients presented with leukopenia. By 12 weeks, white blood cell counts had normalized in 38 patients (88.37%). After 52 weeks, 73.68% of patients showed normalized white blood cell counts. The mean white blood cell count rose from 2.34 ± 0.71*10^9/L at baseline to 6.50 ± 2.84 *10^9/L at 12 weeks, 5.55 ± 2.23 *10^9/L at 24 weeks, and 4.58 ± 1.71 *10^9/L at 52 weeks, all significantly higher than baseline (*P* < 0.0001) (**Figure 4A**). Thrombocytopenia was observed in 45 patients at baseline. At 12 weeks, platelet counts had normalized in 24 patients (53.33%). After 52 weeks, 66.67% of patients had achieved normal platelet counts. Nine patients had platelet counts below 30 *10^9^/L at baseline; during follow-up, platelet counts fully recovered in five patients and rose above 30 *10^9^/L in three. Besides, the mean platelet count increased from 58.47 ± 28.82 *10^9/L at baseline to 137.2 ± 70.70 *10^9/L at 12 weeks, 132.1 ± 63.70 *10^9/L at 24 weeks, and 133.5 ± 63.92 *10^9/L at 52 weeks (*P* < 0.0001) (**Figure 4B**). Among these patients, fourteen received granulocyte colony-stimulating factor (G-CSF), and three received recombinant human interleukin-11 for injection.

### Glucocorticoid dose

The glucocorticoid dose (prednisone or equivalent) was significantly reduced from baseline (39.98 ± 10.09 mg/d) to 18.35 ± 7.87 mg/d at 12 weeks, 9.752 ± 4.72 mg/d at 24 weeks, and 5.69 ± 2.95 mg/d at 52 weeks (*P* < 0.0001) (**Figure 4C**). The proportion of patients receiving a prednisone dose ≤ 7.5mg/d increased from 1.44% at baseline to 10.07% at 12 weeks, 41.58% at 24 weeks, and 76.19% at 52 weeks (**Figure 4D**). At 52 weeks, 5 patients (7.94%) discontinued glucocorticoid.

### Safety

All adverse events, including incidence and severity, were recorded. Serious adverse events were defined as those resulting in hospitalization, prolonged hospital stay, disability, impaired work ability, life-threatening, death, or congenital malformation. Adverse events were recorded in 28 patients (20.14%). The most common event was upper respiratory tract infection (10.79%), followed by urinary tract infection (5.04%), with no serious adverse events observed (**Table 3**).

**Table 3:** Adverse events that occurred during the period of telitacicept treatment.

Adverse events	Telitacicept (n=139)
Number of patients with adverse events, n (%)	28 (20.14)
Upper respiratory tract infection	15 (10.79)
Urinary tract infection	7 (5.04)
Injection site reactions	3 (2.16)
Rash	2 (1.44)
Herpes virus infection	1 (0.72)

## Discussion

### Global disease activity and steroid sparing

SLE is a systemic autoimmune disease involving multiple systems and organs, characterized by recurrent flares and remissions along with extensive autoantibody production. Inadequate disease control can lead to irreversible organ damage and premature mortality. Current treatment guidelines recommend a treat-to target approach for SLE, aiming for clinical remission or a lupus low disease activity state (LLDAS) (9) (14). Researches have shown that achieving LLDAS in the early stage of SLE treatment can significantly reduce organ damage (15–17). Another systematic review reported that patients who attained remission or LLDAS had substantially lower risks of organ damage accrual compared to non-responders, with hazard ratios (HR) ranging from 0.49 to 0.75 for remission and 0.19 to 0.88 for LLDAS. Thus, maintaining LLDAS or remission is associated with reduced risks of organ damage, disease flares, severe infections, hospitalizations, and overall mortality (18). Although conventional regimes comprising glucocorticoids and immunosuppressants can partially control disease activity, a substantial proportion of patients fail to achieve LLDAS. According to data from the Chinese SLE Treatment and Research group, only 0.76% of Chinese SLE patients achieve the DORIS definition of “off-drug remission”, 2.47% achieve “clinical remission”, and 7.85% attain a state of “low disease activity” (19). Additionally, the prolonged use of conventional therapies is often limited by severe cumulative toxicities, including osteoporosis, cardiovascular events, infection, malignancy, teratogenicity, and premature ovarian failure. Thus, there is a critical, unmet clinical need for novel, targeted therapies that can provide superior efficacy with a more favorable safety.

### Role of B cells and telitacicept in SLE

Aberrant proliferation and dysregulated B cell signaling are closely related to the pathogenesis of SLE. Many studies have demonstrated that B cells secrete autoantibodies, which form immune complexes with self-antigens. These complexes deposit in tissues and activate the complement system, triggering complement-dependent cytotoxicity and subsequent tissue damage (20). Furthermore, autoantibodies recruit effector cells including macrophages, neutrophils, and natural killer cells through the antibody-dependent cellular cytotoxicity and Fc receptors to mediate tissue damage (21). Additionally, B cells serve as antigen-presenting cells expressing MHC class II molecules, interacting with T cells to drive SLE progression (22). B cells produce numerous cytokines to mediate immune inflammation. For instance, secretion of interferon-γ and interleukin-6 promotes germinal center survival and T follicular helper cell differentiation (23). These studies suggest the pivotal role of B cells in the immunologic cascade of SLE. Members of the tumor necrosis factor superfamily, specifically BAFF and APRIL, are key regulators of B cell immune response. At present, there are several B cell-targeted therapies for SLE. Telitacicept effectively inhibits the abnormal proliferation and differentiation of B cells, thereby rapidly controls the progression of SLE. In a Phase 2b clinical trial published in 2023, 249 patients with active SLE were randomized to receive either telitacicept or placebo for 48 weeks; the results showed that the proportion of patients achieving an SRI-4 response was significantly higher with telitacicept than with placebo (24). Similarly, a Phase 3 trial involving 335 patients with active SLE demonstrated a higher clinical response rate with telitacicept compared to placebo over 52 weeks of treatment alongside standard care. However, there were also higher rates of upper respiratory tract infections, decreased immunoglobulin levels, and injection-site reactions in the telitacicept group (13). Therefore, although the efficacy of telitacicept is promising, further studies are needed to clarify its benefits and risks in real-world clinical practice.

### Efficacy in disease activity and immune parameters

This real-world study aimed to investigate the efficacy and safety of telitacicept in patients with SLE. This study is a retrospective, uncontrolled and observational study. Compared with Phase 2b (24) and Phase 3 (13) clinical trials, this study enrolled patients with active SLE, without excluding severe lupus nephritis and lupus central nervous system disease, which can better reflect the efficacy of telitacicept in the real world. A total of 139 patients who received telitacicept for at least 3 months were included in this study, of whom 101 patients were treated for 6 months to 1 year and 63 patients were treated for more than 1 year. Our results showed that SLEDAI-2K scores at 12 weeks, 24 weeks, and 52 weeks of treatment were 4.626 ± 3.652, 2.416 ± 2.984, and 1.476 ± 2.583, respectively, which were significantly lower than those at baseline (11.42 ± 4.679). Particularly, the decrease in SLEDAI-2K scores was more pronounced in the subgroup with severe active disease (baseline SLEDAI-2K > 12). The percentage of patients with reduction of ≥ 4 points in SLEDAI-2K score at 52 weeks was 87.30%, which was slightly higher than the percentage at 48 weeks reported in the Phase 2b study (75.8 to 79%) (24) and higher than the percentage of reduction of ≥ 4 points in SELENA-SLEDAI at 52 weeks reported in the Phase 3 study (70%) (13). These findings suggest that telitacicept has a significant effect in clinical practice. Additionally, immunological indicators were also improved. C3 and C4 levels were significantly increased, while the positive rate of anti-dsDNA was decreased. The levels of IgG, IgA, and IgM were significantly lower than baseline but still within the normal range. These observations indicated that telitacicept exerts effective immunosuppressive role in the early stage of treatment.

### Renal outcomes

We also focused on SLE-related renal abnormalities. 74 patients (53%) were diagnosed with lupus nephritis. After 12, 24, and 52 weeks of telitacicept treatment, 24-hour urinary protein decreased to 1.12 ± 1.41 g/24h, 0.42 ± 0.46 g/24h, and 0.39 ± 0.64 g/24h, respectively, all significantly lower than the baseline (1.59 ± 1.68 g/24h). UACR progressively decreased, while serum albumin level gradually increased. Serum albumin level was improved from 33.29 ± 8.30 (g/L) at baseline to 40.99 ± 4.91(g/L) at 12 weeks, 44.09 ± 3.64 (g/L) at 24 weeks and 44.11 ± 3.32 (g/L) at 52 weeks. Renal function indicators, including creatinine and glomerular filtration rate, also improved. While previous studies have reported the efficacy of telitacicept in patients with class III-V lupus nephritis (25, 26), our findings further corroborate the clinical benefit of telitacicept when combined with conventional therapies in the treatment of lupus nephritis.

### Hematologic outcomes

Currently, reports on the treatment of SLE with hematological involvement remain limited. Xuefei Li reported a case of immune thrombocytopenia (ITP) secondary to SLE, which was successfully controlled with telitacicept after prior treatment with dexamethasone, cyclosporine, and hetrombopag (27). In our cohort, 67 patients (48.2%) presented with hematological system damage, including leukopenia and thrombocytopenia. After treatment with telitacicept, white blood cells and platelets were significantly increased compared with baseline. Notably, the baseline platelet count of 4 patients was less than 10*10^9/L, and 3 of them returned to normal after treatment, while the remaining patient maintained a platelet count between 30-49*10^9/L. These findings indicated that telitacicept is also effective in managing SLE-associated leukopenia and thrombocytopenia.

### Glucocorticoid dosage reduction outcomes

Long-term glucocorticoid therapy is associated with numerous adverse effects. The 2023 EULAR recommendations for the management of SLE suggested that glucocorticoids should be reduced to a maintenance dose of ≤5 mg/day (prednisone equivalent) (10). The definition of LLDAS requires a glucocorticoid dosage of ≤7.5 mg/day (prednisone or equivalent). In our study, the prednisone dose was significantly reduced after combination therapy with telitacicept, and the proportion of patients with prednisone ≤7.5 mg/day reached 76.19% at 52 weeks, which was higher than the percentage reported in the Phase 3 study (44.9%) (13). Moreover, at 52weeks, 5 patients (7.94%) had completely discontinued glucocorticoid therapy. These findings indicate that telitacicept combined with conventional treatment offers a steroid-sparing potential in a heterogeneous real-world cohort.

At the same time, we need to emphasize the importance of concomitant standard therapies. Hydroxychloroquine was used in 94.96% of the patients and immunosuppressive were used in 89.21% of the patients at baseline. Most patients continued to receive hydroxychloroquine and immunosuppressive during the treatment, which indicated that concomitant standard treatment with telitacicept is important to control disease activity, reduce glucocorticoid dose and improve organ functions.

### Safety

Furthermore, this study demonstrated a favorable safety profile for telitacicept. A total of 28 adverse events occurred in the whole study, mainly consisting of upper respiratory tract infection and urinary tract infection, which was consistent with the results of Phase 2b (24) and Phase 3 (13) clinical trials, and the incidence was lower. No serious adverse events occurred. These results indicated that telitacicept is well-tolerated in clinical use and does not increase the risk of serious infection.

### Limitations and future directions

This study also has several limitations. First, disease activity was assessed using only SLEDAI-2K score without PGA score, making it impossible to evaluate LLDAS attainment rates. Second, this was a single-center, retrospective, uncontrolled study with a relatively small sample size, primarily due to strict inclusion criteria requiring at least three months of telitacicept treatment and complete follow-up data. The absence of a control group prevents direct efficacy comparisons between telitacicept and conventional therapies or other biological agents. Therefore, future large-scale, long-term, comprehensive, controlled observational studies should be carried out to further evaluate the efficacy and safety of telitacicept in real-world settings.

## Conclusion

In conclusion, this real-world study indicated that telitacicept combined with conventional therapy was associated with improvements in disease activity and organ functions in the treatment of SLE with favorable safety.

## Data Availability

The original contributions presented in the study are included in the article/**Supplementary Material**. Further inquiries can be directed to the corresponding authors.
